# Myosin VI Reduces Proliferation, but Not Differentiation, in Pluripotent P19 Cells

**DOI:** 10.1371/journal.pone.0063947

**Published:** 2013-05-17

**Authors:** Takeshi Takarada, Miki Kou, Noritaka Nakamichi, Masato Ogura, Yuma Ito, Ryo Fukumori, Hiroshi Kokubo, Gabriela B. Acosta, Eiichi Hinoi, Yukio Yoneda

**Affiliations:** 1 Laboratory of Molecular Pharmacology, Division of Pharmaceutical Sciences, Kanazawa University Graduate School, Kanazawa, Ishikawa, Japan; 2 Instituto de Investigaciones Farmacológicas (ININFA), CONICET-UBA, Buenos Aires, Argentina; Sanford-Burnham Medical Research Institute, United States of America

## Abstract

**Background:**

We have previously shown marked upregulation of the mRNA and corresponding protein for the cellular motor molecule myosin VI (Myo6) after an extremely traumatic stress experience, along with a delayed decrease in 5-bromo-2′-deoxyuridine incorporation in the murine hippocampus, a brain structure believed to undergo adult neurogenesis. In this study, we investigated the role of Myo6 in both proliferation and differentiation in pluripotent P19 cells by using stable transfection and RNA interference techniques.

**Methodology/Principal Findings:**

Stable overexpression of Myo6 not only led to significant inhibition of the reducing activity of 3-(4,5-dimethylthiazol-2-yl)-2,5-diphenyl-2H-tetrazolium bromide (MTT) and the size of clustered aggregates in P19 cells, but also resulted in selectively decreased mRNA expression of the repressor type proneural gene Hes5 without affecting the expression of neuronal and astroglial marker proteins. In P19 cells transfected with Myo6 siRNA, by contrast, a significant increase was found in the size of aggregate and MTT reduction along with increased Sox2 protein levels, in addition to marked depletion of the endogenous Myo6 protein. In C6 glioma cells, however, introduction of Myo6 siRNA induced a drastic decrease in endogenous Myo6 protein levels without significantly affecting MTT reduction. The Ca^2+^ ionophore A23187 drastically increased the luciferase activity in P19 cells transfected with a Myo6 promoter reporter plasmid, but not in HEK293, Neuro2A and C6 glioma cells transfected with the same reporter.

**Conclusions/Significance:**

These results suggest that Myo6 may play a predominant pivotal role in the mechanism underlying proliferation without affecting differentiation to progeny lineages in pluripotent P19 cells.

## Introduction

We have previously shown significant alterations in endogenous levels of both glutamic and gamma-aminobutyric acids in particular brain structures after the extremely traumatic water immersion restraint stress (WIRS) experience in rats [1]. In mice with WIRS, a variety of long-lasting bidirectional behavioral abnormalities similar to symptoms in patients with posttraumatic stress disorder (PTSD) are seen even 14 days after the traumatic stress, proceeded by marked and transient deterioration in incorporation of the thymidine analog 5-bromo-2′-deoxyuridine (BrdU) in the dentate gyrus (DG) of the hippocampus [2].

The hippocampal DG has a unique ability to generate new neurons throughout the entire life of an individual, often referred to as adult neurogenesis [3], [4]. The newly born cells develop into granule neurons and are capable of extending axonal projections to the CA3 area [5], [6]. In our previous study, in fact, WIRS and subsequent flashback experiences similarly lead to drastic but transient decreases in the numbers of both neuronal and astroglial cells derived from neural progenitors, with suppression of proliferation in the DG in mice [7]. Moreover, the extremely stressful experience induced a rapid but transient increase in the expression of both the mRNA and corresponding protein for the cellular motor molecule Myosin VI (Myo6) in the mouse hippocampus [7].

Myosin VI (Myo6) is an actin-based molecule responsible for the transport of intracellular cargos including proteins, vesicles and organelles [8], [9]. Myo6 has a unique property to move to the pointed (minus) ends of actin filaments, which is favorable for orientation toward the cell center, whereas other native myosin molecules are able to migrate toward the barbed (plus) ends of F-actin [10]. This unique directionality allows Myo6 localized at the plasma membrane to pull endocytic vesicles inward using the migration force generated by its movement on F-actin [11]. In addition, Myo6 exhibits a unique biological function through several binding partners, such as disable-2 [12], G-Alpha Interacting Protein (GAIP) C-terminus-interacting protein-1 [13], [14], synapse-associated protein-97 [15] and RNA polymerase II [16].

These previous studies led us to propose a possible role of Myo6 in mechanisms underlying the aforementioned suppression of BrdU incorporation into the hippocampal DG enriched with neural progenitor cells, in mice with traumatic stress. Therefore, the present series of experiments was intended to examine the role of Myo6 in mediating proliferation for self-replication and differentiation into progeny lineages using murine embryonal carcinoma P19 cells endowed to proliferate and differentiate into neuronal and astroglial lineages in the presence of all-*trans* retinoic acid (ATRA) as a model for neural progenitor cells [17].

## Materials and Methods

### P19 Cell Culture

Mouse embryonal carcinoma P19 cells (ATCC, Manassas, VA, USA) were cultured in alpha minimal essential medium (αMEM) (GIBCO BRL, Grand Island, NY, USA) supplemented with 10% fetal bovine serum (FBS) (GIBCO BRL). P19 cells are able to differentiate into a neural lineage in the presence of ATRA (Sigma, St. Louis, MO, USA) as described previously [18]. Undifferentiated P19 cells were routinely sub-cultured at least three times at intervals of 2 days prior to the treatment with ATRA. P19 cells were then cultured in bacterial-grade Petri dishes in the presence of 0.5 µM ATRA for 4 days under floating conditions, trypsinized for dispersion, and plated onto tissue culture-grade dishes coated with poly-L-lysine (Sigma) for induction of spontaneous differentiation during culture in the absence of ATRA for 12 days under adherent conditions. The cultures were always maintained in a humidified atmosphere of 5% CO_2_ and 95% air at 37°C with a medium change every 2 days.

### Establishment of Stable Transfectants

The full-length coding region of porcine Myo6, which was kindly donated by Dr. Tama Hasson (UCSD, San Diego, CA, USA), was inserted into pSI vector using the Ligation High reagent as described previously [19]. Mouse embryonal carcinoma P19 cells were plated at a density of 1.5×10^5^ cells/cm^2^, followed by culture in Dulbecco’s modified Eagle’s medium (DMEM) (GIBCO BRL) with 10% FBS for 24 h and subsequent stable transfection with pSI-Myo6, pSI-GFP and pcDNA3.1 vectors, or pSI, pSI-GFP and pcDNA3.1 vectors, using 2 µg of DNA and Lipofectamine and Plus reagent (Invitrogen, San Diego, CA, USA) in 0.5 ml of Opti-MEM (Invitrogen) as described previously [20]. After 24 h, and every 48 h thereafter for 2 weeks, the culture medium was replaced with fresh medium containing 500 µg/ml of G418 (Invitrogen). Pools of 6 clones were isolated as stable transfectants with either the Myo6 expression vector or empty vector (EV) in P19 cells for further studies using clones between passages of 3 and 6 [21]. BLAST database (http://blast.ncbi.nlm.nih.gov) analysis revealed a 96% homology between porcine and murine Myo6 proteins.

### Determination of mRNA and Protein Expression

Reverse transcription-polymerase chain reaction (RT-PCR) analysis was conducted as described previously [22]. Quantification of PCR products was done by real time-based RT-PCR using an Mx3005P (Agilent Technologies, Santa Clara, CA, USA) with a THUNDERBIRD SYBR qPCR Mix (TOYOBO, Osaka, Japan). The relative amount of each transcript was normalized against glyceraldehyde-3-phosphate dehydrogenase (GAPDH) [23]. The primer sequences used are summarized in Table 1.

**Table 1:** Primers used in this study.

Genes	Upstream (5′-3′)	Downstream (5′-3′)
**Cyclins**		
CyclinA1	AGCGACAGCTACTGAGGATGG	CAGCAACCAAGGAAGGAAGATAC
CyclinA2	GGTGCTGCCAACTGTCAAGAT	AAGTCAGCAGTTTGGTTTGGTTG
CyclinD1	AGTGCGTGCAGAAGGAGATT	CACAACTTCTCGGCAGTCAA
**bHLH transcriptional factor**		
Hes1	CCAGCCAGTGTCAACACGA	AATGCCGGGAGCTATCTTTCT
Hes3	GATACGGAAACGAAAGCTGGA	GAGGCAAGGGTTGAGAACAGA
Hes5	AGTCCCAAGGAGAAAAACCGA	GCTGTGTTTCAGGTAGCTGAC
Mash1	GCAACCGGGTCAAGTTGGT	GTGGTTGGAGTAGTTGGGGG
Math1	CAACGACAAGAAGCTGTCCA	CCTAACTGGCCTCGTCAGAG
Math3	AGGCCAATGCTAGAGAACGA	ATGGTTGAGGCTTGAGATGG
NeuroD1	ATGACCAAATCATACAGCGAGAG	TCTGCCTCGTGTTCCTCGT
Ngn1	CAGTAGTCCCTCGGCTTCAG	GGGTCAGTTCTGAGCCAGTC
**Pluripotentiality marker**		
????	AGAGGATCACCTTGGGGTACA	CGAAGCGACAGATGGTGGTC
Nanog	CACAGTTTGCCTAGTTCTGAGG	GCAAGAATAGTTCTCGGGATGAA
**Others**		
Myo6	CGCAGAACTACGCGATACAA	CCTTGCCAGCTACAAGAAGG
Gapdh	ACCACAGTCCATGCCATCAC	TCCACCACCCTGTTGCTGTA

Western blotting was done by using antibodies against Myo6 (Santa Cruz Biotechnology, Santa Cruz, CA, USA), Doublecortin (Cell Signaling Technology, Danvers, MA, USA), Synapsin I (Merck Ltd., Darmstadt, Germany), neuronal nuclei (NeuN) (Merck Ltd.), microtubules-associated protein-2 (MAP2) (Sigma), glial fibrillary acidic protein (GFAP) (Sigma), Sox2 (Santa Cruz Biotechnology) and β-tubulin (Sigma) as described previously [22].

### Determination of Cellular Proliferation

Five different visual fields each in parallel experiments were chosen at random from each culture well with undifferentiated P19 cells cultured with ATRA for a period of up to 4 days, and examined under a phase contrast micrograph in a blinded fashion, followed by calculation of areas of round aggregates composed of clustered proliferating cells for summation using Scion Image β 4.02 software (Scion Co., Frederick, MD, USA) as described previously [22]. Moreover, cultured cells collected by centrifugation at 300 g were incubated with 0.5 mg/ml 3-(4,5-dimethyl-2-thiazolyl)-2,5-diphenyl-2H-tetrazolium bromide (MTT) (Sigma) in phosphate-buffered saline (PBS) for 1 h at 37°C, followed by addition of 0.04 M HCl in isopropanol and subsequent shaking of the mixture for 10 min to dissolve the formazan. The dissolved suspension was scanned by an ELISA reader for measurement of the absorbance at a wavelength of 550 nm as described previously [24].

### Determination of Cellular Viability

Stable transfectant cells were cultured in the presence of ATRA for 4 days, followed by re-plating on dishes previously coated with poly-L-lysine for further culture for 2 h and subsequent double staining for DNA with 10 µg/ml Hoechst33342 (Sigma) and 5 µg/ml propidium iodide (PI) (Sigma). Cells were observed under a confocal microscope (LSM-510; Carl Zeiss, Jena, Germany). The number of cells stained with these dyes was individually counted in five different visual fields selected at random in a blinded fashion in order to calculate the percentages of dead cells stained with the membrane-impermeable dye PI out of the total cells stained with the membrane-permeable dye Hoechst33342. Cellular viability was also quantified by the activity of lactate dehydrogenase (LDH) released into the culture medium by an enzyme-coupled color development method as descried previously [25] with minor modifications [22].

### Determination of Cellular Differentiation

Pluripotent P19 cells have been shown to differentiate into neuronal and astroglial lineages after commitment in the presence of ATRA as described previously [17]. Undifferentiated P19 cells were plated onto 0.2% agarose coated dishes (φ100 mm) at a density of 1×10^5^ cells/ml in αMEM supplemented with 5% FBS and 0.5 µM ATRA, followed by culture for 4 days under floating conditions favorable for proliferation. These floating cells were harvested and trypsinized, followed by plating onto dishes previously coated with poly-L-lysine at a density of 2×10^5^ cells/ml in αMEM supplemented with 10% FBS. They were subsequently cultured for an additional period up to 6 days in the absence of ATRA to induce spontaneous differentiation as shown with murine fetal cortical progenitors [22]. The culture medium was changed every 2 days, and cultures were maintained in a humidified atmosphere of 5% CO_2_/95% air at 37°C.

### Preparation of Reporter Plasmids

A reporter plasmid with the Myo6 promoter was prepared as follows. The mouse Myo6 promoter was obtained by cloning using the forward primer 5′-ACGCGT (MluI site) GCAAGAACCCTCACTGGC-3′ and the reverse primer 5′-CTCGAG (XhoI site) AGGCTGCCGGGCTGCGGGCG-3′ from the mouse genome. The Myo6 (-1086 to+210) promoter fragment was cloned into the promoterless pGL-3 basic vector (Promega, Madison, WI, USA), to create the recombinant plasmid –1086/+210 Myo6-Luc.

### Reporter Assay

Pluripotent P19 cells were transiently transfected with a reporter plasmid containing the promoter region of Myo6 (−1086 bp to +210 bp) along with the internal control vector pRL-SV40 (Promega) using the Lipofectamine 2000 reagent. Transfected cells were plated at 1×10^5^ cells/ml in plates (φ100 mm) previously coated with 0.2% agarose, and cultured in αMEM supplemented with 10% FBS and 0.5 µM ATRA. Twenty-four hours after transfection of the reporter plasmid, the cells were exposed to 1 µM A23187 (Sigma) for 6 h, followed by a medium change, and then cultured for an additional 24 or 48 h. The cells were then lysed for determination of luciferase activity using specific substrates in a luminometer according to the manufacturer’s protocol. Transfection efficiency was normalized against the activity of pure Renilla luciferase [22]. Approximately 30% of cells expressed green fluorescent protein (GFP) in P19 cells transfected with the EGFP-C2 plasmid under the transfection conditions employed.

### Data Analysis

Quantitative data are expressed as the mean ± S.E. and the statistical significance was determined by the two-tailed Student’s *t*-test or one-way analysis of variance (ANOVA) with the Bonferroni/Dunnett *post hoc* test.

## Results

### Stable Transfection of Myo6

**Figure 1:**
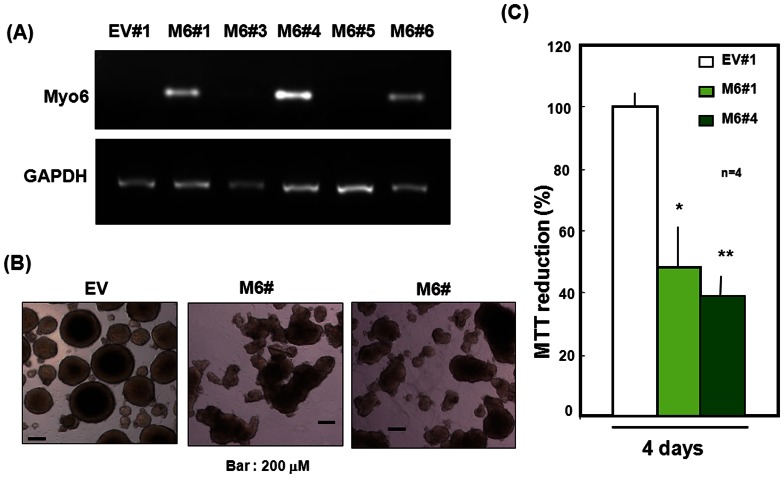
Stable overexpression of Myo6. (A) Mouse embryonal P19 cells were stably transfected with Myo6 expression vector, followed by selection by G418 and subsequent determination of Myo6 mRNA expression with RT-PCR analysis. Several clones of stable transfectants were cultured with ATRA for 4 days for subsequent (B) micrographic observation and (C) MTT reduction determination. Each value represents the mean ± S.E. in 4 different experiments. *P<0.05, **P<0.01, significantly different from the control value in stable EV transfectants.

**Figure 2:**
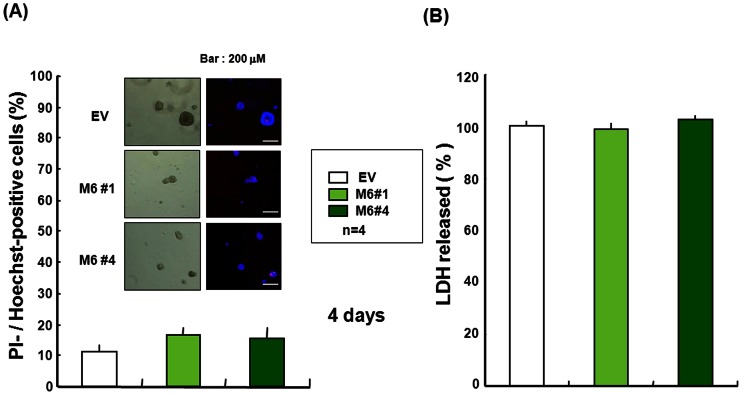
Cellular viability in stable transfectants of P19 cells. Several clones of stable transfectants were cultured with ATRA for 4 days, followed by determination of (A) the percentage of PI-positive cells over Hoechst33342-positive cells and (B) the activity of LDH released into culture medium. Each value represents the mean ± S.E. in 4 different experiments.

In our previous study [26], transient overexpression of Myo6 led to a decrease in the size of clustered aggregates composed of proliferating cells during culture with ATRA in pluripotent P19 cells. Amongst 6 clones selected by G418 screening in pluripotent P19 transfectants, Myo6 mRNA was strongly detected in several clones, such as M6#1 and M6#4, upon RT-PCR analysis (Figure 1A). These transfectants were invariably clustered, forming round aggregates while continuing to grow in size up to 4 days during culture under floating conditions in the presence of ATRA, along with increased proliferation. However, stable Myo6 transfection led to a marked decrease in the size of clustered aggregates formed after culturing with ATRA for 4 days (Figure 1B). In both stable Myo6 transfectant clones, in fact, a significant deterioration was seen in MTT reducing activity compared to that in stable EV transfectants after 4 days in culture (Figure 1C). In both stable Myo6 transfectants, after 4 days, no significant changes were detected in the percentages of dead cells stained with PI out of the total cells stained with Hoechst33342 (Figure 2A) or the release of LDH into the culture medium (Figure 2B) compared with stable EV transfectants.

### Expression of Proliferation-relevant Markers

**Figure 3:**
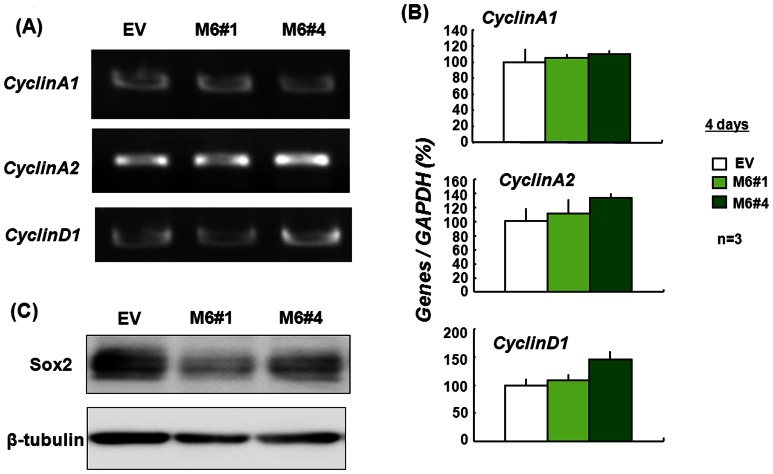
Expression of cell cycle regulator mRNA and progenitor marker protein in stable transfectants. Stable transfectants were cultured with ATRA for 4 days, followed by determination of expression of (A,B) mRNA with RT-PCR and (C) protein with Western blotting. Quantitative data are shown as the mean ± S.E. in 3 different experiments.

To evaluate the mechanism underlying the possible deterioration in proliferative activity, each stable transfectant clone was screened for mRNAs for several cell cycle regulators of the Cyclin family [27]. However, no marked alteration was found in mRNA expression of CyclinA1, CyclinA2 or CyclinD1 between stable Myo6 and EV transfectants (Figure 3A). Densitometric quantification of these data confirmed the absence of significant changes in the mRNA expression of these cyclins in the stable Myo6 transfectants examined (Figure 3B), in contrast to the suppressed proliferation seen after Myo6 overexpression. We then examined expression of the progenitor marker protein Sox2 in stable Myo6 transfectants cultured for 4 days in the presence of ATRA under floating conditions. In both stable Myo6 transfectant clones, despite having lower MTT reduction activity than control clones, a marked decrease was seen in the endogenous Sox2 protein level (Figure 3C).

**Figure 4:**
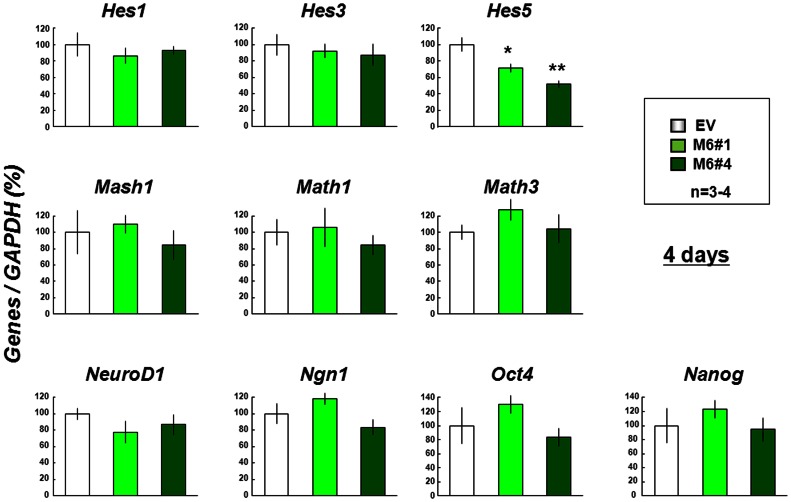
Expression of mRNA for bHLH proneural transcription factors and pluripotentiality markers in stable transfectants. Stable transfectant cells were cultured with ATRA for 4 days, followed by determination of mRNA expression levels with real time-based RT-PCR. *P<0.05, **P<0.01, significantly different from each control value in stable EV transfectants.

An attempt was thus made to determine whether stable overexpression of Myo6 leads to altered expression of a variety of basic helix-loop-helix (bHLH) genes responsible for the positive and negative regulation of proliferation for self-renewal and/or neuronal differentiation in undifferentiated neural stem cells [28]. We examined quantitative expression profiles of both activator [Mash1, Math1, Math3, NeuroD1 and Neurogenin1 (Ngn1)] and repressor [hairy and enhancer of split-1 (Hes1), Hes3 and Hes5] classes of bHLH proneural genes, in addition to pluripotentiality markers such as Oct4 and Nanog, via real time RT-PCR. In undifferentiated clones of stable Myo6 transfectants, among the different genes tested, statistically significant downregulation was only found in Hes5 mRNA compared to that in stable control transfectants (Figure 4).

### Marker Protein Expression

**Figure 5:**
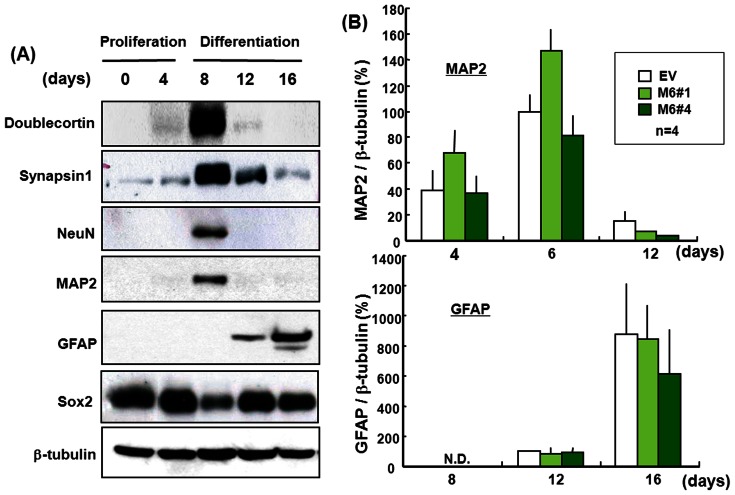
Expression of several marker proteins in stable transfectants. (A) Pluripotent P19 cells were cultured with ATRA for 4 days under floating conditions, followed by dispersion of clustered aggregates and subsequent further culture in the absence of ATRA for an additional 12 days under adherent conditions. Cells were harvested different days during culture for determination of several marker protein expression with Western blotting analysis. (A) Typical pictures are shown with similar results in 3 separate determinations. (B) Stable transfectants were cultured for a period of up to 16 days as described above, followed by quantitative determination of MAP2 and GFAP on Western blotting. Each value represents the mean ± S.E. in 4 different experiments.

P19 cells were cultured in the presence of ATRA for 4 days under floating conditions to induce clustered aggregate formation due to self-replication, followed by immunostaining for the neural progenitor cell marker nestin. Cells clustered in round aggregates were immunoreactive for nestin and also exhibited a marked increase in MTT reduction, both indices of cellular proliferation [26]. The aggregated cells were then dispersed and cultured further in the absence of ATRA for an additional 12 days under adherent conditions. Four days after replating, the cells were observed to express neuronal marker proteins such as MAP2 and NeuN at high levels without expressing the astroglial marker protein GFAP, followed by disappearance of MAP2-positive cells and subsequent appearance of cells immunoreactive for GFAP 8 to 12 days after re-plating (Figure 5A). By contrast, levels of the other neuronal marker Synapsin 1 were highest on Day 8 with progressively lower expression on Day 12, Day 14 and Day 16 during their 16 days in culture. Moreover, the migrating neuronal progenitor marker Doublecortin was strongly expressed by cells cultured for 8 days, whereas the progenitor marker Sox2 was expressed in cells throughout the 16 days in culture. Similar expression profiles were seen for immunoreactive MAP2 and GFAP in stable EV transfectants during the 16 days, while no significant difference was found in the temporal expression profile of either MAP2 (Figure 5B, upper panel) or GFAP (Figure 5B, lower panel) in the stable Myo6 transfectants M6#1 and M6#4 compared to those cells with EV.

### Knockdown of Myo6 Expression with siRNA

**Figure 6:**
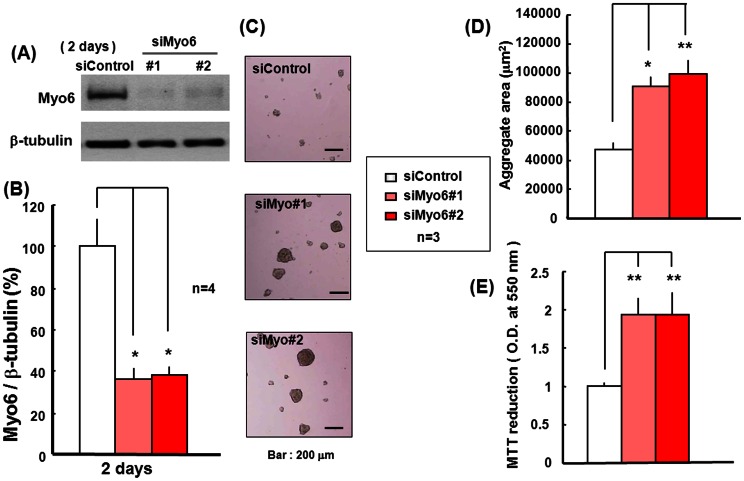
Knockdown of Myo6 protein expression with siRNA in P19 cells. P19 cells were transfected with Myo6 siRNA in the presence of ATRA for 2 days, followed by determination of endogenous levels of Myo6 protein on Western blotting. Typical pictures are shown in the panel (A), while quantitative data are shown in the panel (B). Cells were also subjected to (C) phase contrast micrographic observation, (D) aggregate size measurement and (E) MTT reduction determination. Each value represents the mean ± S.E. in 3–4 different experiments. *P<0.05, **P<0.01, significantly different from each control value obtained in cells transfected with siControl.

We next examined the effects of Myo6 knockdown on proliferation and differentiation of pluripotent P19 cells using RNA interference techniques. Cells were transfected with Myo6 siRNA, followed by culture with ATRA for 2 days under floating conditions and subsequent determination of endogenous levels of Myo6. A drastic decrease in endogenous Myo6 protein levels in cells transfected with 2 different Myo6 siRNA oligonucleotides compared with those with a control oligonucleotide was invariably seen on Western blotting (Figure 6A). Quantification analysis confirmed a significant decrease in Myo6 protein levels in P19 cells treated with Myo6 siRNA (Figure 6B). In the cells with decreased endogenous Myo6 levels, a statistically significant increase was found in the sizes of the clustered aggregates composed of proliferating cells (Figure 6C) (Figure 6D). Similarly, both Myo6 siRNA oligonucleotides were effective in significantly increasing MTT reduction in P19 cells cultured for 2 days (Figure 6E).

**Figure 7:**
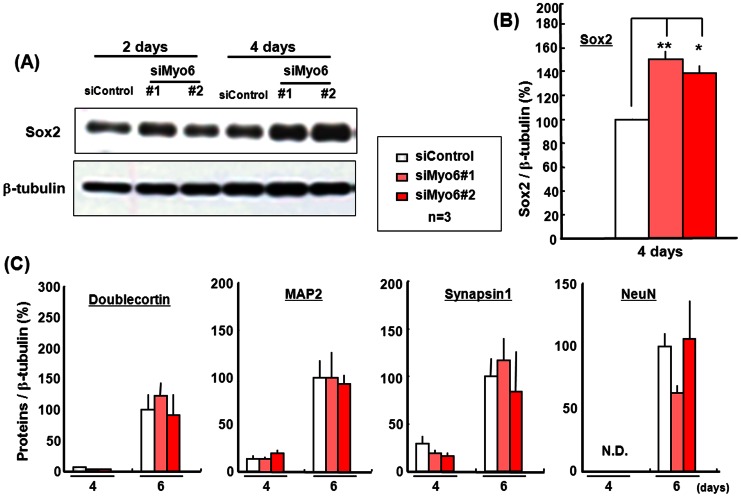
Expression of several marker proteins in P19 cells with Myo6 siRNA. (A) Pluripotent P19 cells were transfected with Myo6 siRNA in the presence of ATRA for 2 or 4 days under floating conditions, followed by determination of Sox2 protein expression with Western blotting analysis. Typical pictures are shown in the panel (A), while quantitative data are shown in the panel (B) for cells cultured for 4 days. (C) Cell aggregates were also dispersed and further cultured in the absence of ATRA for an additional 2 days under adherent conditions for determination of several marker protein expression with Western blotting analysis. Each value represents the mean ± S.E. in 3 different experiments. *P<0.05, **P<0.01, significantly different from each control value obtained in cells transfected with siControl.

We then examined the expression of Sox2 in P19 cells treated with Myo6 siRNA for 2 or 4 days in the presence of ATRA under floating conditions. Western blotting analysis clearly revealed a marked, statistically significant increase in endogenous Sox2 protein levels in P19 cells transfected with Myo6 siRNA for 2 and 4 days compared to those exposed to control siRNA (Figure 7A) (Figure 7B). Cells were next transfected with Myo6 siRNA for 4 days with ATRA, followed by dispersion of the aggregates and further culturing in the absence of ATRA for an additional 2 days under adherent conditions to promote spontaneous differentiation. A marked increase was invariably seen in endogenous levels of several neuronal marker proteins, including Doublecortin, MAP2, Synapsin 1 and NeuN, in P19 cells after the removal of ATRA and subsequent additional culture, irrespective of transfection with Myo6 siRNA, while Myo6 knockdown failed to significantly affect endogenous levels of these neuronal marker proteins during culture for an additional 2 days (Figure 7C).

### Cell Selectivity

**Figure 8:**
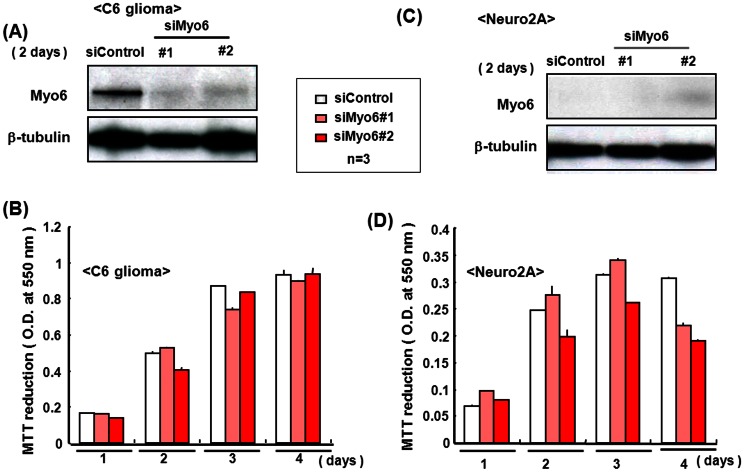
Myo6 protein expression in C6 glioma and Neuro2A cells with siRNA. C6 glioma and Neuro2A cells were transfected with Myo6 siRNA, followed by culture for 2 days under individually appropriate conditions and subsequent determination of endogenous levels of Myo6 protein on Western blotting. Cells were also cultured for different periods up to 4 days toward determination of MTT reduction. Typical pictures are shown with Myo6 expression by C6 glioma cells in the panel (A) and that by Neuro2A in the panel (C), while quantitative data are shown with MTT reduction by C6 glioma cells in the panel (B) and that by Neuro2A cells in the panel (D), respectively. Each value represents the mean ± S.E. in 3 different experiments.

To test the selectivity of the correlation between Myo6 expression and cellular proliferation, the MTT assay was used to determine whether Myo6 siRNA affects cellular proliferation in cultured proliferating cells other than pluripotent P19 cells. In C6 glioma cells cultured for 2 days, marked expression of Myo6 protein was seen in a manner sensitive to knockdown by siRNA (Figure 8A). Although MTT reduction gradually increased in C6 glioma cells treated with control siRNA during increasing culture periods up to 4 days, Myo6 siRNA failed to significantly affect MTT reduction during the 4 days (Figure 8B). In the neuronal cell line Neuro2A, moreover, Myo6 protein was not highly expressed irrespective of transfection with Myo6 siRNA (Figure 8C). Furthermore, Myo6 siRNA again failed to significantly modulate MTT reduction in Neuro2A cells cultured for 4 days (Figure 8D).

**Figure 9:**
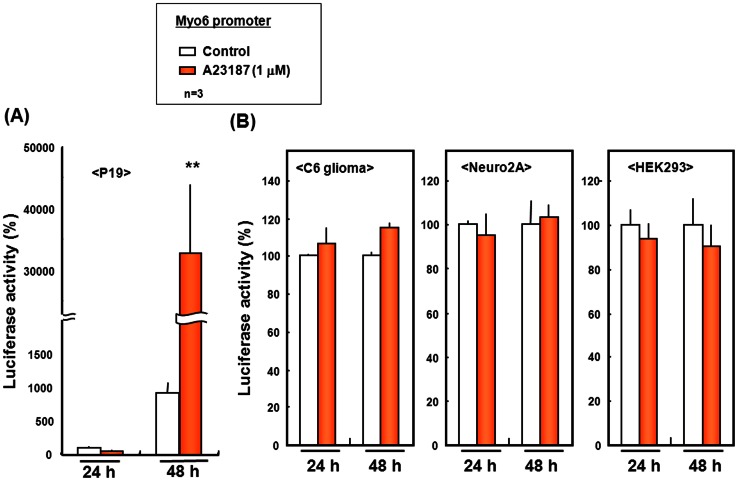
Myo6 promoter activity in different cells exposed to A23187. (A) Pluripotent P19 cells, and (B) astroglial C6 glioma, neuronal Neuro2A and peripheral HEK293 cells were individually transfected with the Myo6 reporter plasmid for 24 h, followed by exposure to 1 µM A23187 for 6 h and subsequent further culture for an additional 2 or 4 days for consequential determination of the luciferase activity. Each value represents the mean ± S.E. in 3 different experiments. **P<0.01, significantly different from each control value obtained in cells not exposed to A23187.

We have previously shown a more than twofold increase in Myo6 mRNA expression is induced in P19 cells exposed to the calcium ionophore A23187 [26]. In fact, in P19 cells bearing a luciferase reporter plasmid linked to the Myo6 promoter region (−1086 to +210 bp), 1 µM A23187 drastically increased luciferase activity determined 48 h after transfection with the reporter plasmid (Figure 9A). By contrast, A23187 was ineffective in significantly increasing luciferase activity in C6 glioma, Neuro2A and HEK293 cells transfected with the reporter plasmid at 1 µM when determined 24 and 48 h after transfection (Figure 9B).

## Discussion

The essential importance of the present findings is that proliferation was markedly suppressed in pluripotent P19 cells stably overexpressing the motor protein Myo6 without induction of cell death. Conversely, the current findings that proliferation was significantly promoted in P19 cells by decreased endogenous Myo6 levels due to siRNA transfectionargue in favor of the idea that Myo6 negatively regulates self-replication in pluripotent P19 cells endowed to either proliferate for self-renewal or differentiate into neuronal and astroglial lineages. Taking into consideration the unresponsiveness of different cell cycle regulators and bHLH proneural genes other than Hes5, it is conceivable that downregulation of Hes5 expression is at least in part responsible for the inhibition of proliferation in stable Myo6 transfectants. In mice defective for the activator bHLH factor Mash1, for example, severe loss of neuronal precursors together with disappearance of the Notch signaling target Hes5 is seen in the ventral telencephalon normally enriched for Mash1 during neurogenesis [29]. Notch signaling is shown to mediate a cell-cell interaction required for the maintenance of actively dividing cells and subsequent generation of different progeny cell lineages in a manner dependent on expression of the repressor type bHLH genes Hes1 and Hes5, whichantagonize activator type neuronal bHLH genes such as Mash1 [30]. By contrast, both Hes1 and Hes5 are essential Notch effectors in negatively regulating mammalian neuronal differentiation [31].

However, the reason why Myo6 overexpression inhibited cellular proliferation without affecting the mRNA levels of several cyclins in P19 cells is still not clear. A future comprehensive analysis should be conducted on mRNA expression by all cyclin family members and relevant kinases to evaluatethe possible involvement of particular cyclin family members in the underlying mechanisms other than those tested here. Although the activator type bHLH factor NeuroD1 plays a pivotal role in the survival and neuronal differentiation of neural stem cells during adult neurogenesis [32], [33], NeuroD1 is also identified as a gene highly correlated with the terminal differentiation into neurons during postnatal and adult neurogenesis on microarray gene analysis [34]. To our knowledge, this is the first direct demonstration of predominant inhibition of proliferation without affecting commitment and/or differentiation into progeny lineages through a mechanism relevant to downregulation of the repressor bHLH proneural gene Hes5 in undifferentiated pluripotent P19 cells with stable Myo6 overexpression.

Nevertheless, the exact signaling pathway from stable overexpression of Myo6 to preferential downregulation of Hes5 amongst different repressor and activator bHLH proneural genes examined is not clarified so far. One possible but hitherto unproven speculation is that intracellular Myo6 overexpression promotes the migration of particular cargo proteins and/or organelles required to suppress the transactivation of the Hes5 gene toward the nucleus. For instance, the mammalian target of rapamycin (mTOR) signals are known to be essential for maintenance of neural progenitor status through a mechanism associated with upregulation of Hes5 and Pax6 expression in pluripotent P19 cells cultured with ATRA [35]. The prevailing view is that activation of the mTOR signaling pathway is required for protein translation, cell growth and deteriorated autophagy toward the facilitation of cell growth and proliferation [36]. These previous findings all gave rise to an idea that stable overexpression of Myo6 would lead to negative regulation of the mTOR signaling pathway responsible for subsequent promotion of gene transactivation of particular activator and repressor bHLH factors in neural progenitors. The reason why downregulation of Hes5 expression failed to affect subsequent differentiation fates in pluripotent P19 cells, however, is not clear. A future analysis of overexpression and knockdown of Hes5 in P19 cells will undoubtedly give a clue to this question.

In hippocampal neurons from *sv*/*sv* mice defective for Myo6, a significant deficit is seen in internalization of a particular ionotropic subtype of non-N-methyl-D-aspartate receptor (NMDAR) after stimulation [37]. Both Myo6 and its binding protein are required for glutamate release mediated by the interaction of brain-derived neurotrophic factor with TrkB receptors [38]. In addition to the selective and rapid expression of Myo6 in response to an acute traumatic stressful experience [7], flashbacks to virtual re-experience have been demonstrated to induce Myo6 protein expression in the murine hippocampus [26]. Synaptic abnormalities occur along with short dendritic spines and marked astrogliosis in the hippocampus of Myo6-null mice, while profound synaptic loss is seen in cultured hippocampal neurons with dominant-negative disruption of Myo6, in addition to those prepared from Myo6-deficient mice [37]. These previous findings clearly give support to the importance of Myo6 in molecular mechanisms underlying synaptogenesis in the hippocampus. The possibility that abnormally overexpressed Myo6 could lead to a variety of hippocampal dysfunctions seen after a traumatic stress experience, which would at least in part relate to the pathogenesis of PTSD in human beings [39], is not ruled out.

Although Myo6 is highly expressed in cultured astrocytes expressing GFAP rather than in cultured neurons expressing NeuN [26], a previous study showed localization of Myo6 to neurons in association with postsynaptic density [37]. However, immunoreactive Myo6 expression is not localized in particular neuronal layers within the cerebral cortex, striatum, hippocampus or olfactory bulb in the mouse brain [7]. The inconsistent distribution profiles between Myo6 and GFAP in discrete brain regions, along with temporal expression profiles in pluripotent P19 cells [26], do not argue in favor of the possible predominance of Myo6 expression in certain types of cells in the brain. In fact, Myo6 was originally demonstrated to be responsible for clathrin-mediated endocytosis and vesicular trafficking in non-neuronal cells [11], [40]. The fact that activation of NMDAR leads to a significant increase in Myo6 mRNA expression in pluripotent P19 cells expressing functional NMDAR channels [26] gives support to an idea that traumatic stress induces glutamate release [41], [42] for suppression of self-replication through induction of Myo6 in response to activation of NMDAR expressed by neural progenitor cells in the adult mouse DG. Indeed, prior blockade of NMDAR by dizocilpine prevents decreased incorporation of BrdU into the DG as well as behavioral abnormalities in mice exposed to WIRS [2].

### Conclusion

It thus appears that the motor protein Myo6 selectively inhibits cellular proliferation required for self-replication through a mechanism relevant to downregulation of the repressor bHLH proneural factor Hes5 in undifferentiated P19 cells. Further evaluation of Myo6 expression will be undoubtedly beneficial for the future discovery and development of drugs useful for the prophylaxis, therapy and treatment of a variety of abnormalities and malfunctions seen in patients suffering from different neurodegenerative and neuropsychiatric diseases.
